# Improving physical movement during stroke rehabilitation: investigating associations between sleep measured by wearable actigraphy technology, fatigue, and key biomarkers

**DOI:** 10.1186/s12984-024-01380-3

**Published:** 2024-05-28

**Authors:** Madeleine J. Smith, Michael Pellegrini, Brendan Major, Marnie Graco, Stephanie Porter, Sharon Kramer, Katherine Sewell, Sabrina Salberg, Zhibin Chen, Richelle Mychasiuk, Natasha A. Lannin

**Affiliations:** 1 Department of Neuroscience, School of Translational Medicine, Monash University, 99 Commercial Road, Melbourne, VIC 3004 Australia; 2 Alfred Health, Melbourne, VIC 3004 Australia; 3 Austin Health, Melbourne, VIC 3084 Australia

**Keywords:** Stroke Rehabilitation, Rehabilitation, Sleep, Fatigue, Actigraphy, Biomarkers, Inflammation

## Abstract

**Background:**

Sleep disturbance and fatigue are common in individuals undergoing inpatient rehabilitation following stroke. Understanding the relationships between sleep, fatigue, motor performance, and key biomarkers of inflammation and neuroplasticity could provide valuable insight into stroke recovery, possibly leading to personalized rehabilitation strategies. This study aimed to investigate the influence of sleep quality on motor function following stroke utilizing wearable technology to obtain objective sleep measurements. Additionally, we aimed to determine if there were relationships between sleep, fatigue, and motor function. Lastly, the study aimed to determine if salivary biomarkers of stress, inflammation, and neuroplasticity were associated with motor function or fatigue post-stroke.

**Methods:**

Eighteen individuals who experienced a stroke and were undergoing inpatient rehabilitation participated in a cross-sectional observational study. Following consent, participants completed questionnaires to assess sleep patterns, fatigue, and quality of life. Objective sleep was measured throughout one night using the wearable Philips Actiwatch. Upper limb motor performance was assessed on the following day and saliva was collected for biomarker analysis. Correlation analyses were performed to assess the relationships between variables.

**Results:**

Participants reported poor sleep quality, frequent awakenings, and difficulties falling asleep following stroke. We identified a significant negative relationship between fatigue severity and both sleep quality (*r*=-0.539, *p* = 0.021) and participants experience of awakening from sleep (*r*=-0.656, *p* = 0.003). A significant positive relationship was found between grip strength on the non-hemiplegic limb and salivary gene expression of Brain-derived Neurotrophic Factor (*r* = 0.606, *p* = 0.028), as well as a significant negative relationship between grip strength on the hemiplegic side and salivary gene expression of C-reactive Protein (*r*=-0.556, *p* = 0.048).

**Conclusion:**

The findings of this study emphasize the importance of considering sleep quality, fatigue, and biomarkers in stroke rehabilitation to optimize recovery and that interventions may need to be tailored to the individual. Future longitudinal studies are required to explore these relationships over time. Integrating wearable technology for sleep and biomarker analysis can enhance monitoring and prediction of outcomes following stroke, ultimately improving rehabilitation strategies and patient outcomes.

**Supplementary Information:**

The online version contains supplementary material available at 10.1186/s12984-024-01380-3.

## Introduction

Active participation in motor rehabilitation programs is critical after stroke, which emphasizes the importance of understanding factors that enable individuals to engage fully in the rehabilitation process. Following stroke, neuroscience research has demonstrated that the severity of cortical damage [1, 2], inflammatory response to stroke [3], genetic factors [4], circadian disruption [5], and depression and anxiety [6, 7] may affect movement outcomes. While the field of physical rehabilitation has also shown that, in the clinic, the severity of stroke [8], sleep disruption [9–15], fatigue [16–21], inflammation [22–24], depression and anxiety [25, 26], and the presence of comorbidities [27–29], all coalesce to affect both engagement in rehabilitation and motor recovery. To date, however, there has been little acknowledgement that many of these factors may be influenced by the rehabilitation hospital environment. The disruption of medical patients in acute wardrooms has previously been shown [30], but there is little research investigating the relationships between sleep and motor function in the setting of inpatient rehabilitation wards. Given the pivotal role of inpatient rehabilitation in the stroke recovery pipeline, understanding the impact of these factors, inclusive of hospital environment, on sleep, fatigue, and movement outcomes is key.

The impact of each of these factors (predominantly independent of one another) has previously been discussed, albeit most commonly in community living stroke populations. For instance, post-stroke fatigue is experienced by 25 to 85% of stroke survivors [31], and is influenced by sleep disturbances [32] as well as physical activity and fitness [33], although the relationship between fatigue and objective measures of motor performance and strength remain unclear [34]. Measures of post-stroke mood also correlate significantly with self-reported fatigue [18, 35, 36]. Additionally, stroke survivors with increased circulating interleukin (IL)-1β and c-reactive protein (CRP) have higher levels of post-stroke fatigue [37–39]. Further, brain-derived neurotrophic factor (*BDNF)* allele variations are indicative of stroke survivors motor function [40]. Although the relationship between salivary gene expression and motor function during inpatient rehabilitation has not been specifically investigated, biomarkers are a potential non-invasive screening target. Objective biomarkers offer potential insights into the mechanisms of post-stroke fatigue during motor rehabilitation. The opportunity for research to shape clinical care has been further strengthened by our recent ability to monitor the features of sleep outside of the laboratory environment, permitting a real-time understanding of the impact of the hospital ward environment on sleep parameters.

There have been no previous studies to objectively measure sleep during inpatient rehabilitation and assess its association with motor function. Therefore, the primary aim of this study was to investigate the impact of sleep quality on motor function in people with stroke, by capturing subjective and objective measures of sleep using wearable technology during inpatient rehabilitation. We hypothesized that reduced sleep quality would have a negative impact on motor function following stroke. Additionally, we aimed to determine if there were correlations between fatigue, sleep and motor function during inpatient rehabilitation. Investigating the relationship between post-stroke sleep and objective biomarkers as predictors of motor recovery following stroke is an important step towards developing personalized rehabilitation programs [41]. Therefore, we aimed to determine if salivary biomarkers of stress, inflammation, and neuroplasticity were associated with fatigue or motor function post-stroke.

## Methods and materials

### Study design and procedure

In this cross-sectional, single-site observational study, all participants were screened against eligibility criteria and were invited to participate. Following written consent, demographic data and data pertaining to each participant’s stroke (including stroke date, severity, and type as well as National Institutes of Health Stroke Scale (NIHSS) and Functional Independence Measure (FIM™) scores) were extracted from the electronic medical record prior to clinical assessment. Additionally, participants completed standardized questionnaires (see below) to assess their quality of life, fatigue, and sleep patterns following stroke. Participants wore a sleep monitoring device overnight, known as an actigraph, and reported their recollection of sleep on the following day. After the actigraph was removed, participants completed motor assessments and saliva was collected for gene expression analysis. Thus, there were two assessment sessions scheduled across consecutive days. All measurements were taken during inpatient rehabilitation at Caulfield Hospital, a standalone rehabilitation facility based in metropolitan Melbourne, Australia. The study was conducted in accordance with the Declaration of Helsinki and the protocol was approved by the Alfred Health Human Research Ethics Committee (Project ID: 660/21) and Monash University.

### Participants

Eighteen individuals with stroke who were receiving inpatient rehabilitation participated in the study. Individuals were eligible for the study if they had a diagnosis of stroke of no greater than 3-months prior, were aged ≥ 18 years, able to provide written, informed consent in English, and were identified as having upper limb motor impairment by their treating occupational therapist. Participants with pre-existing motor impairments of any cause were excluded. The participants entered the service between December 2021 and June 2022.

### Questionnaires

Participants completed the EuroQol EQ-5D-5L [42] to capture their perceived self-rating of health-related quality of life across the dimensions of mobility, self-care, usual activities, pain/discomfort, and anxiety/depression. The nine-item Fatigue Severity Scale (FFS-9) [43] was used to measure fatigue and its relationship to motivation, exercise, physical functioning, duties and responsibilities, disabling symptoms, and work, family or social life, alongside the Visual Analog Fatigue Scale (VASF) [44] to provide a global rating of fatigue (scale 0–10). Participants also completed the Leeds Sleep Evaluation Questionnaire [45] to capture perceived sleep patterns (with the reference for the person back to their pre-stroke sleep); which consists of 10 questions relating to sleep latency, quality of sleep, awakening from sleep, and behavior following wakefulness.

#### Actigraphy and sleep diary

Sleep was monitored for one night. Participants wore the Philips Actiwatch Spectrum 2 (Philips Respironics, Pittsburgh, PA, USA) on their wrist. The Actiwatch was placed on the participant’s wrist on the hemiparetic side [46]. The Actiwatch software (Actiware version 6.0, Philips Respironics, OR, USA) automatically determined sleep onset and offset times via pre-determined activity thresholds. Within the lights off/lights on times, sleep onset time was classified as the first minute of a 10-minute immobile period with < 2 activity counts in any 30-s period. Ten consecutive minutes of activity was defined as sleep offset. During sleep, activity threshold counts > 40 per 30-s epoch were defined as awake. This allowed calculation of the number of awakenings and amount of awake time. Sleep efficiency was calculated as the percentage of time spent in bed asleep relative to the total time spent in bed between getting into bed and getting up the following morning [46].

Participants were also asked to complete a sleep diary to document the time they got into bed with the intention to sleep, the approximate time it took for them to fall asleep, the time they awoke the following morning, and the number of times they awoke overnight. These data were then used to calculate sleep onset latency and total sleep time. Nursing observations were collected from charts including overnight functional activities (e.g. toileting).

### Clinical assessment

On the day that immediately followed sleep monitoring, participants underwent motor testing by a physiotherapy assessor and saliva samples were collected for telomere length and gene expression analysis.

Upper extremity motor performance was measured via the Box and Block test [47], and is reported as blocks per second. Hemiplegic and non-hemiplegic upper extremity grip strength was measured as a maximum voluntary contraction using a Jamar dynamometer and reported as kg [48].

Saliva was collected immediately prior to clinical assessment of motor performance using the passive drool method by Oragene-DNA self-collection kits (DNAGenotek, Canada) consisting of a collection tube and a capture straw. Thirty minutes prior to collection, participants were instructed to refrain from drinking, eating and taking medication. RNA was extracted from saliva samples using the Allprep DNA/RNA Mini Kit (Cat# 80,204; Qiagen, Germany), according to manufacturer’s protocols. Quality and quantity were measured using the NanoDrop 2000 (Thermo Fisher Scientific, USA). Two micrograms of RNA were reverse transcribed to complementary DNA (cDNA) with qScript™ XLT cDNA SuperMix (Quantabio, USA) for downstream quantitative real-time polymerase chain reaction (qRT-PCR). For the following genes: *NR3C1, CRP, IL1-*$\beta$, *TNF-*$\alpha$, *BDNF, MTNR1A, MTRN1B*, each sample was run in duplicate on a 96-well plate, with 1 x SYBR Green FastMix ROX, 0.5$\mu$M of forward and reverse primers, and 10ng of cDNA on the CFX Connect-Real-Time PCR Detection system (BioRad, USA). The ${2}^{-\varDelta \varDelta CT}$ method was used for analysis, with the housekeeping genes *Ywhaz* and *Cyca* used for normalization as previously described [49, 50]. For telomere length, DNA was extracted from saliva samples using the QIAamp DNA Mini Kit (Cat# 51,306; Qiagen, Germany), according to the manufacturer’s protocols. Quality and quantity were measured using the NanoDrop 2000 (Thermo Fisher Scientific, USA). DNA was diluted to 20ng/$\mu$l with TE buffer and used for downstream qRT-PCR analysis. All samples were run in duplicate for both TEL and 36B4 primers with 1 x SYBR Green FastMix ROX on the CFX Connect-Real-Time PCR Detection system (BioRad, USA), as previously described [51, 52]. All primers were obtained from IDT, with cycling parameters and primer sequences detailed in Supplementary File 2.

### Statistical analysis

Statistical analyses were conducted using SPSS (Version 28.0, IL, USA). Shapiro-Wilk test was conducted to assess the normality of continuous variables [53]. Pearson’s correlation coefficient was used to assess the relationships between variables that follow approximately normally distributed distributions, and Spearman’s rank correlation coefficient was calculated for variables that do not [54]. Point biserial correlation was used to measure relationships between dichotomous variables and the continuous variables [54]. The strength of the correlation between two variables were categorized as follows; poor (< 0.50), moderate (0.50–0.75), good (0.75–0.90) and excellent (> 0.90) [55]. All statistical tests were performed at the 5% significance level (*p* = 0.05).

## Results

### Participant characteristics

The characteristics of the 18 participants included in this study are shown in Table 1. As expected within an inpatient rehabilitation environment, most of the participants had experienced a stroke of moderate severity according to their NIHSS. Hospital admission data indicated that seven participants had a speech or language problem and had difficulty understanding instructions or had major speech and language impairments (aphasia). Additionally, the participants experienced moderate cognitive deficits across various domains, including comprehension, expression, social interaction, problem solving, and memory. The majority of the participants reported no or minimal anxiety or depression.

**Table 1:** Characteristics of participants. Values listed as mean ± SD or proportions (%)

Characteristic	Total sample (*n* = 18)
Age (yrs)	72 ± 10
Sex, number male (%)	12 (67%)
Stroke type, number ischemic (%)	14 (78%)
Days since stroke	31 ± 22
Stroke volume (cm^3^)	131 ± 237
Stroke region	
Brain stem, n (%)	7 (39%)
Basal ganglia, n (%)	3 (17%)
Temporal/parietal lobes, n (%)	3 (17%)
MCA territory, n (%)	2 (11%)
Cerebellum, n (%)	1 (6%)
Multi-territorial cerebrum, n (%)	1 (6%)
Internal capsule, n (%)	1 (6%)
Stroke severity *(*NIHSS*)*	
No stroke symptoms, n (%)	1 (6%)
Minor stroke, n (%)	5 (28%)
Moderate stroke, n (%)	10 (56%)
Moderate-severe stroke, n (%)	1 (6%)
Severe stroke, n (%)	0 (0%)
Depression and anxiety (EQ-5D-5 L)	
No/slight symptoms (score 1–2)	14 (78%)
Moderate symptoms (score 3)	4 (22%)
Severe/extreme symptoms (score 4–5)	0 (0%)
Cognitive function, FIM (cognitive score 0–35)	18 ± 6
Motor function, FIM (motor score 0–91)	38 ± 18
Motor performance (grip strength), kg	
Hemiplegic	16.3 ± 12.2
Non-hemiplegic	24.4 ± 12.7
Motor performance (box and block test), blocks per min	
Hemiplegic	15.3 ± 11.6
Non-hemiplegic	27.8 ± 7.0

### Sleep in stroke patients during inpatient rehabilitation

Participant’s sleep during inpatient rehabilitation is summarized in Table 2. The Philips Actiwatch was an objective measure of participants’ sleep and results indicated that participants slept for an average of 8.4 h (SD = 1.6), they woke an average of 25 times (SD = 14) throughout the night, and their sleep efficiency was 84.5% (SD = 10.8). Subjective reporting by participants indicated that 7 participants awoke during the night for toileting (38.7%), 3 participants woke for spontaneous or unknown reason (16.7%), 3 participants woke for nursing or related care (16.7%), and 2 participants woke to hospital noises (11.1%). Overnight sleep-related nursing notations were recorded for 8 participants, with half (*n* = 4) reporting that the participant “slept well”.

**Table 2:** Assessment of post-stroke sleep quality via objective (Philips Actiwatch, nursing progress notes) and subjective (patient reported awakening) measurements. Values listed as mean ± SD or proportions (%)

Measurement	
Philips Actiwatch (*n* = 18)	
Sleep onset latency (mins)	31.1 ± 40.7
Total sleep time (hrs)	8.4 ± 1.6
Number of awakenings (count)	25 ± 14
Wake after sleep (mins)	30.3 ± 22.9
Sleep efficiency (%)	84.5 ± 10.8
Nursing progress notes reporting (*n* = 8)	
Slept well (%)	4 (50%)
Disrupted overnight (%)	2 (25%)
Woke for toilet (%)	1 (12.5%)
Disturbed/agitates (%)	1 (12.5%)
Patient report awakening (*n* = 18)	
Toilet (%)	7 (38.9%)
Spontaneous/unknown (%)	3 (16.7%)
Nursing care related (%)	3 (16.7%)
Hospital noises (%)	2 (11.1%)
No reports (%)	3 (16.7%)

Half of all participants (*n* = 9) indicated that getting to sleep was “more difficult” following stroke compared to before (defined as a score of 0–4/10), the majority (67%, *n* = 12) indicated that they were “more restless” than usual (defined as a score of 0–4/10), and 44% (*n* = 8) had more difficulty awakening. Of interest, 56% (*n* = 10) indicated that they were clumsier upon awakening following stroke compared to before (defined as a score of 0–4/10).

### The relationship between sleep and motor performance during inpatient rehabilitation

The relationships between motor performance, and both subjective (Leeds Sleep Evaluation Questionnaire) and objective (Philips Actiwatch) measures of sleep, are shown in Table 3. There was a negative relationship between motor performance measured via grip strength on the non-hemiplegic side and awakenings from sleep (*r*=-0.48, *p* = 0.046). Additionally, there was a positive relationship between sleep onset latency and motor performance measured via the box and block test on the non-hemiplegic side (*r* = 0.743, *p* = < 0.001). There were no significant relationships between hemiplegic limb performance and objective or subjective measures of sleep.

**Table 3:** Relationship between post-stroke sleep and motor function

Motor test	Leeds sleep evaluation questionnaire					Philips Actiwatch				
	Going to sleep ‘*r*’	Quality of sleep ‘*r*’	Awakening from sleep ‘*r*’	Behavior following awakening ‘*r*’	Total ‘*r*’	Sleep onset latency ‘*r*’	Total sleep time ‘*r*’	Number of awakenings ‘*r*’	Wake after sleep ‘*r*’	Sleep efficiency ‘*r*’
Grip strength, kgs										
Hemiplegic	-0.22	-0.08^†^	-0.35	-0.19	-0.30	0.201	-0.309	-0.109	0.091	-0.242
Non-hemiplegic	-0.26	-0.22^†^	-0.48*	0.01	-0.33	0.348	-0.173	0.087	-0.113	-0.237
Box and block test, blocks per s										
Hemiplegic	-0.14	0.22^†^	0.13	0.04	0.02	-0.222	-0.226	0.118	0.442	-0.165
Non-hemiplegic	-0.24	0.12^†^	-0.32^†^	0.11^†^	-0.25^†^	0.743*	-0.356	-0.288	-0.58	-0.269

‘*r*’ indicates Pearson correlation coefficient. ^†^ indicates Spearman rank correlation coefficient for data violating normality. ^*^ indicates significant correlation between post-stroke sleep quality and the independent variable (*p* < 0.05).

### The relationship between fatigue and motor function

**Fig. 1:**
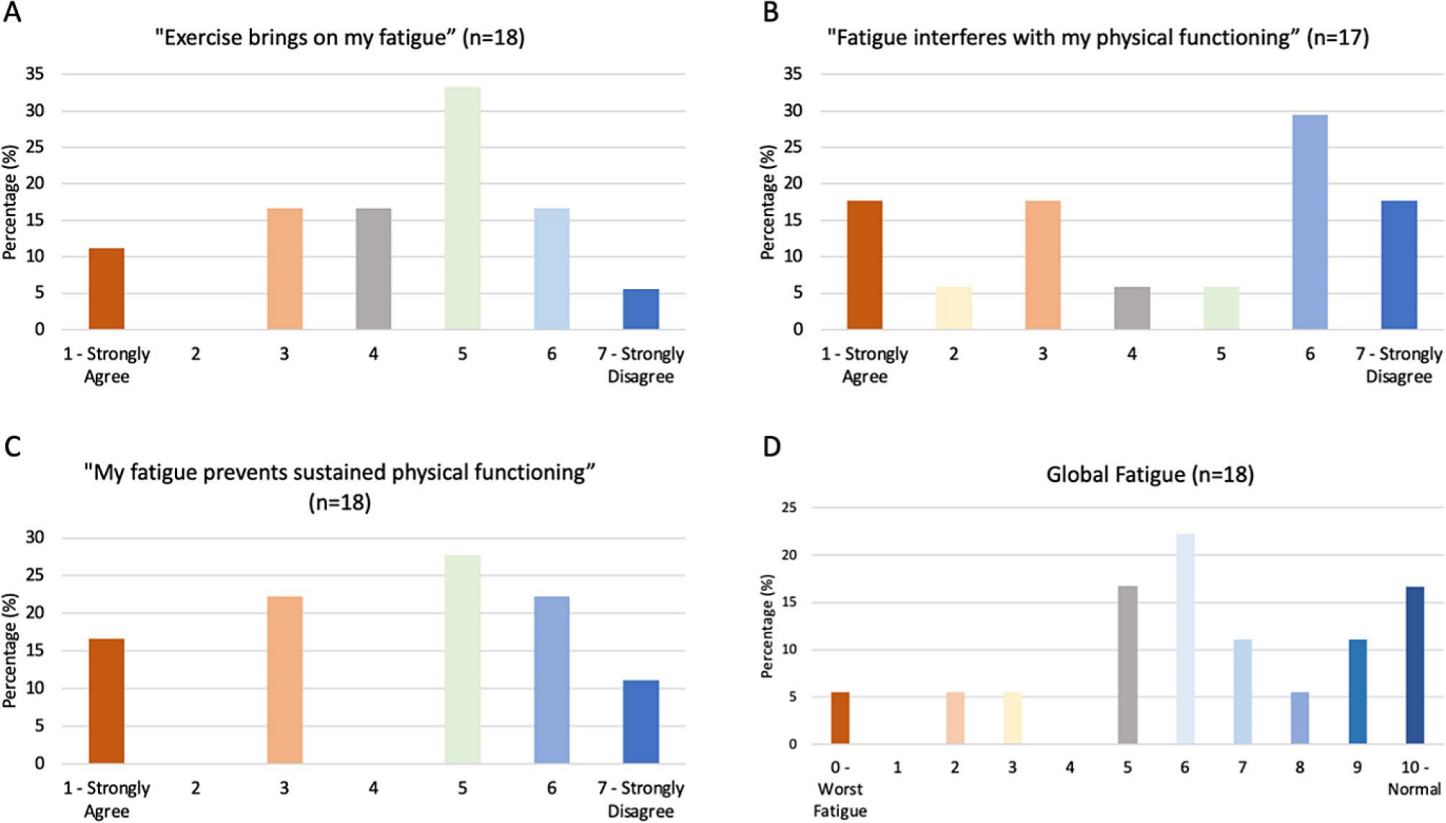
Participants experience of fatigue measured via the (**A**-**C**) fatigue severity scale and the (**D**) visual analogue fatigue scale

Although there was no significant correlation between fatigue and motor function (Supplementary File 1), 28% (*n* = 5) of participants indicated that exercise brings on their fatigue (defined as a score of 1–3/7) (Fig. 1A). Additionally, 41% (*n* = 7) indicated that fatigue interferes with their physical functioning (defined as a score of 1–3/7) (Fig. 1B). 39% (*n* = 7) of participants indicated that sustained physical functioning is prevented by fatigue (defined as a score of 1–3/7) (Fig. 1C). 39% of participants (*n* = 7) experienced moderate global fatigue (defined as a score of 5–6/10), measured on a Visual Analogue Fatigue Scale (Fig. 1D).

### The relationship between sleep and fatigue

There was a significant negative correlation between the severity of fatigue (Fatigue Severity Scale) and the experience of participants when they woke up (*r*=-0.656, *p* = 0.003) (Table 4). Additionally, there was a significant negative correlation between the severity of fatigue and the total score for participants evaluation of their sleep (*r*=-0.539, *p* = 0.021) (Table 4).

**Table 4:** Relationship between post-stroke sleep and fatigue

Measure of fatigue	Leeds sleep evaluation questionnaire					Philips Actiwatch				
	Going to sleep ‘*r*’	Quality of sleep ‘*r*’	Awakening from sleep ‘*r*’	Behavior following awakening ‘*r*’	Total ‘*r*’	Sleep onset latency ‘*r*’	Total sleep time ‘*r*’	Number of awakenings ‘*r*’	Wake after sleep ‘*r*’	Sleep efficiency ‘*r*’
Fatigue severity scale	-0.273	-0.337^†^	-0.258	-0.656*	-0.539*	-0.078	0.255	0.045	-0.060	0.136
Visual analogue fatigue severity scale	0.03	-0.073^†^	0.179	0.355	0.213	0.06	-0.27	0.186	0.285	-0.359

‘*r*’ indicates Pearson correlation coefficient. ^†^ indicates Spearman rank correlation coefficient for data violating normality. ^*^ indicates significant correlation between post-stroke sleep quality and the independent variable (*p* < 0.05)

### The relationship between salivary biomarkers and motor function

Salivary samples were used to measure gene expression of stress, inflammation, circadian, and neuroplasticity markers. The relationship between gene expression and stroke characteristics, fatigue, and motor performance are summarized in Table 5. There was a positive correlation between the days since stroke and salivary *CRP* gene expression (*r* = 0.615, *p* = 0.025). Additionally, there was a positive relationship between stroke severity and *IL1-β* gene expression (*r* = 0.78, *p* = 0.003). There was no statistical significance between stroke characteristics and telomere length, *NR3C1, TNF-α, BDNF, MTNR1A*, or *MTNR1B* expression. There was a positive relationship between global fatigue and salivary *MTNR1B* expression (*r* = 0.564, *p* = 0.045), but there was no significant relationship between fatigue and other salivary gene expression levels.

**Table 5:** Relationship between motor performance, and salivary gene expression

Expression of gene or telomere length	Stroke volume ‘r’	Stroke severity (NIHSS category) ‘r’	Days since stoke ‘r’	Fatigue measured via FSS ‘r’	Global fatigue measure via VAFS	Grip strength Hemiplegic	Grip strength Non -Hemiplegic	Box and block test Hemiplegic	Box and block test Non-Hemiplegic
Stress									
Telomere length	0.267	0.058	-0.1	-0.234^†^	0.53^†^	-0.052	0.291	0.113	-0.003
NR3C1 gene	0.139	-0.163	0.415^†^	0.133	0.155	-0.482	-0.393	-0.031	-0.094
Inflammation									
CRP gene	0.1	0.455	0.615*	0.212^†^	0.438^†^	-0.556*	-0.187	-0.514	-0.097
IL1-β gene	0.297	0.78*	0.042^†^	-0.185	0.131	0.005	0.378	-0.414	0.153
TNF-α gene	0.448	0.516	0.202^†^	0.127	0.405	-0.266	0.081	-0.266	0.148
Neuroplasticity									
BDNF gene	0.02	0.333	-0.047^†^	0.003	-0.132	0.471	0.608*	-0.043	0.112
Melatonin receptor									
MTNR1A	0.512	0.560	0.546^†^	-0.080	0.423	-0.494	-0.074	-0.491	-0.101
MTNR1B	0.521	0.520	0.313^†^	-0.239	0.564*	-0.558*	-0.026	-0.264	-0.127

‘*r*’ indicates Pearson correlation coefficient. ^†^ indicates Spearman rank correlation coefficient for data violating normality. ^*^ indicates significant correlation between participant stroke characteristics and salivary biomarkers (*p* < 0.05)

There was a negative relationship between grip strength in the hemiplegic side and salivary *CRP* gene expression (*r*=-0.556, *p* = 0.048). Additionally, there was a positive relationship between grip strength on the non-hemiplegic side and salivary *BDNF* gene expression (*r* = 0.608, *p* = 0.028). Furthermore, there was a significant negative relationship between grip strength on the hemiplegic arm and *MTNR1B* salivary gene expression (*r*=-0.558, *p* = 0.048). There were no statistically significant relationships between motor performance and telomere length, or *NR3C1, CRP, IL-1β, TNF-α* or *MTNR1A* gene expression.

## Discussion

The results of this study suggest there is a complex relationship between quality of sleep, fatigue, and motor performance during inpatient rehabilitation. Further, the majority of participants experienced disturbed sleep during inpatient rehabilitation, with our study finding a relationship between this and self-reported fatigue level. Markers of inflammation, neuroplasticity, and melatonin receptor expression could be used as biomarkers to predict outcomes such as fatigue and functional recovery following stroke and should be further investigated in a larger cohort study. The salivary biomarkers with predictability potential in this study included: *CRP, IL1-β, BDNF*, and *MTNR1B* gene expression. By integrating the non-invasive and objective sleep measurement capabilities of Philips Actiwatch technology with biomarker collection, there is potential to personalize and enhance inpatient rehabilitation in hospital settings.

While our study was not able to determine the cause of sleep disturbances, subjective and objective measures of sleep indicate that participants experienced frequent interruptions to their sleep within the hospital environment. Our results support previous findings that hospital environments do disrupt sleep patterns and reduce quality of sleep in adults [56, 57], and that there is a relationship between sleep and fatigue after stroke [34]. While no direct relationship was found between hemiplegic motor function and sleep quality, there were indications that sleep latency and self-reported awakenings were associated with non-hemiplegic dexterity (performance on Box and Block Test) and strength (grip strength) in our sample. Given the acknowledged role that sleep plays on physical performance as well as in recovery from brain injuries [58–60], further research is needed to investigate the complex relationship between fatigue, sleep quality, and rehabilitation participation after stroke.

Depression and anxiety can also cause, or contribute to, increased fatigue and decreased sleep quality after stroke [61, 62]. Fatigue and depression often coexist [63, 64]; however, fatigue is a (somatic) symptom of depression, which can make it difficult to determine the temporal nature of their relationship [65]. Despite their association, however, fatigue can occur independently from depression following stroke [62, 66]. In this cohort, most participants reported no or minimal symptoms of depression or anxiety, suggesting their fatigue may not have been due to depression or anxiety. Nonetheless, given the impact of depression on recovery outcomes, the presence of mood disorders experienced by stroke survivors is important to consider during rehabilitation. Post-stroke depression has been shown to hinder stroke survivors’ ability and motivation to participate in rehabilitation [67–69]. Additionally, depression is associated with poorer functional recovery [70] and greater dependence in undertaking activities of daily living [71, 72].

Our findings confirm previous work outside of the research field of sleep, that biomarkers of inflammation and stress are potential biomarkers for outcomes following stroke [37, 39]. Given that post-stroke fatigue is considered to be one of the most debilitating symptoms, establishing a connection between fatigue and systemic inflammation could be key to developing effective treatments. The positive relationship between *CRP* gene expression and the number of days since stroke may allude to the fact that that inflammation is ongoing and has deleterious effects over time. This finding aligns with previous studies which have shown that there is an ongoing inflammatory response following stroke [73]. *IL1-β* plays a central role in mediating the inflammatory response following stroke, and preclinical studies demonstrate that increased *IL1-β* was associated with larger infarct size [74]. Interestingly, the levels of circulating *IL1-β* in clinical studies are varied, with some studies reporting no change [75, 76] while others demonstrated increased levels following stroke [77]. One particular study in stroke survivors that identified increased circulating *IL1-β* levels correlated this finding with reduced function measured via Barthel Index scores [78]. Our study shows that increased salivary *IL1-β* gene expression could be a non-invasive biomarker of stroke severity, predictive of future performance of daily activities.

Motor performance was also explored in relation to salivary gene expression. There was a negative relationship between grip strength on the hemiplegic side and *CRP* gene expression. Conversely, grip strength on the non-hemiplegic side displayed a positive relationship with salivary *BDNF* gene expression. Our findings substantiate the role *BDNF* plays in facilitating motor recovery in the unaffected limb, possibly through its involvement in neuronal growth and repair processes [79–82].

### Study limitations

As with single-site cohort studies conducted in the clinical setting, this study has several limitations. First, the sample size was relatively small and data regarding the number of participants assessed for eligibility was not recorded, and therefore generalizability may be limited to the specific population studied. Additionally, due to the small sample size we were unable to include participant characteristics as co-variates in the correlation analyses. As we only measured the experience of sleep and motor function during inpatient rehabilitation at one timepoint per participant, we cannot map motor recovery nor potential of sleep to differ across nights. Being cross-sectional by design, this study could not infer causality or determine the temporal dynamics of these relationships. Future longitudinal studies with larger and more diverse cohorts are warranted to validate and expand upon these findings.

## Conclusions

Poor sleep quality and fatigue was reported at high rates in this cohort of stroke survivors undergoing inpatient rehabilitation. Our findings suggest potential relationships between sleep and fatigue, and fatigue and motor performance. Further research is warranted to explore the relationship between these factors, as well as develop prognostic biomarkers to predict recovery and tailor rehabilitation strategies following stoke. The positive relationship found in our study between *CRP* gene expression and the number of days since stroke suggests that inflammatory processes have deleterious effects over time. This finding highlights the need for longitudinal studies to track not only outcomes, but also mediating factors over time. The use of wearable technology to measure sleep during inpatient rehabilitation, in combination with the collection of non-invasive biomarkers, should be combined in larger studies to advance the ability to monitor and predict personalized outcomes following stroke. This study shows that the stroke experience is varied, and that sleep, fatigue, and motor performance are likely interrelated, providing greater support for developing personalized rehabilitation programs.

### Electronic supplementary material

Below is the link to the electronic supplementary material.

Supplementary Material 1

Supplementary Material 2

## Data Availability

The datasets analysed during the current study are available from the corresponding author on reasonable request.

## Declarations

### Abbreviations

EQ-5D-5 L = EuroQol-5D-5 L; FIM = Functional Independence Measure; MCA = middle cerebral artery; NIHSS = National Institutes of Health Stroke Scale.

### Abbreviations

BDNF = Brain derived neurotrophic factor; CRP = C-reactive protein; FSS = Fatigue Severity Scale; IL1-β = Interleukin 1 beta, MTNR1A = Melatonin receptor 1a, MTNR1B = Melatonin receptor 1b; NIHSS = National Institutes of Health Stroke Scale; NR3C1 = Glucocorticoid receptor; TNF- α = Tumour necrosis factor alpha; VAFS = Visual Analogue Fatigue Scale.
