# Supplementary material for: Improving physical movement during stroke rehabilitation: investigating associations between sleep measured by wearable actigraphy technology, fatigue, and key biomarkers
Source: J Neuroeng Rehabil. 2024 May 28;21:84. doi: 10.1186/s12984-024-01380-3 (PMC11131210; doi:10.1186/s12984-024-01380-3)
Supplement: Supplementary file 1 — Supplementary Material 1 [file 12984_2024_1380_MOESM1_ESM.docx]

Supplementary File 2: Primer sequences and cycling parameters for qRT-PCR salivary gene expression

| **Gene symbol** | **Gene name** | **Primer sequence** | **Tm (°C)** | **Cycling parameters** |
| --- | --- | --- | --- | --- |
| *NR3C1* | Glucocorticoid receptor | (+) agaacagcaacatttgaagggc  (-) aggaatgaatcgtcttctcccg | 60 | 1 cycle 95°C 20 sec 40 cycles 95°C 1 sec 40 cycles Tm°C 20 sec + Melt Curve |
| *CRP* | C-Reactive Protein | (+) attcaggcccttgtatcactgg  (-) acacagtgaaggctttgagagg |  |  |
| *IL1-β* | Interleukin 1 beta | (+) ttcgacacatgggataacgagg  (−) ttgttgctccatatcctgtccc |  |  |
| *TNF-α* | Tumor necrosis factor alpha | (+) tgccccaatccctttattaccc  (−) tccagatgtcagggatcaaagc |  |  |
| *BDNF* | Brain-derived neurotrophic factor | (+) aaaaggcattggaactccca  (−) tgctatccatggtaagggcc |  |  |
| *MTNR1A* | Melatonin receptor 1a | (+) catctttgtggtgagcttagcg  (−) ggtgatgttgaatatggagccg |  |  |
| *MTNR1B* | Melatonin receptor 1b | (+) tgtggtgtttgtgatctttgcc  (-) ggaagttttggttcaagagccc |  |  |
| *TEL* | Telomere | (+) ggtttttgagggtgagggtgagggtgagggtgagggt  (−) tcccgactatccctatccctatccctatccctatcccta | 58.4 | 1 cycle 95°C 3 min 30 cycles 95°C 15 sec 30 cycles Tm°C 2 min + Melt Curve |
