# Supplementary material for: Improving physical movement during stroke rehabilitation: investigating associations between sleep measured by wearable actigraphy technology, fatigue, and key biomarkers
Source: J Neuroeng Rehabil. 2024 May 28;21:84. doi: 10.1186/s12984-024-01380-3 (PMC11131210; doi:10.1186/s12984-024-01380-3)
Supplement: Supplementary file 2 — Supplementary Material 2 [file 12984_2024_1380_MOESM2_ESM.docx]

**Supplementary File 1.** Relationship between fatigue and motor function.

| **Variable** | | **Fatigue measured via FSS** |
| --- | --- | --- |
|  |  | ***‘r’*** |
| Grip Strength | Hemiplegic | 0.16 |
|  | Non-hemiplegic | -0.06 |
| Box and block test | Hemiplegic | 0.01 |
|  | Non-hemiplegic | -0.07^†^ |

^†^ indicates Spearman rank correlation coefficient for data violating normality.
